# SVS-VPR: A Semantic Visual and Spatial Information-Based Hierarchical Visual Place Recognition for Autonomous Navigation in Challenging Environmental Conditions

**DOI:** 10.3390/s24030906

**Published:** 2024-01-30

**Authors:** Saba Arshad, Tae-Hyoung Park

**Affiliations:** 1 Industrial Artificial Intelligence Research Center, Chungbuk National University, Cheongju 28644, Republic of Korea; sabarshad1000@gmail.com; 2 Department of Intelligent Systems and Robotics, Chungbuk National University, Cheongju 28644, Republic of Korea

**Keywords:** convolution features, visual place recognition, semantic segmentation, neural networks

## Abstract

Robust visual place recognition (VPR) enables mobile robots to identify previously visited locations. For this purpose, the extracted visual information and place matching method plays a significant role. In this paper, we critically review the existing VPR methods and group them into three major categories based on visual information used, i.e., handcrafted features, deep features, and semantics. Focusing the benefits of convolutional neural networks (CNNs) and semantics, and limitations of existing research, we propose a robust appearance-based place recognition method, termed SVS-VPR, which is implemented as a hierarchical model consisting of two major components: global scene-based and local feature-based matching. The global scene semantics are extracted and compared with pre-visited images to filter the match candidates while reducing the search space and computational cost. The local feature-based matching involves the extraction of robust local features from CNN possessing invariant properties against environmental conditions and a place matching method utilizing semantic, visual, and spatial information. SVS-VPR is evaluated on publicly available benchmark datasets using true positive detection rate, recall at 100% precision, and area under the curve. Experimental findings demonstrate that SVS-VPR surpasses several state-of-the-art deep learning-based methods, boosting robustness against significant changes in viewpoint and appearance while maintaining efficient matching time performance.

## 1. Introduction

Visual place recognition (VPR) serves as a crucial component in mobile robot navigation and localization, essentially functioning as a task akin to content-based image retrieval. The primary objective of VPR is to ascertain whether the visual data currently captured by the robot correspond to a place it has visited before, and if so, to precisely identify which place it is [1].

However, visual place recognition poses formidable challenges due to the expectation that intelligent robots should function autonomously in dynamic environments over extended durations. On one hand, images captured at a specific location can undergo substantial changes over time due to variations in conditions like lighting from day to night, weather, seasonal variations across the year, and shifts in perspective. On the other hand, various distinct places within an environment may exhibit significant visual similarities, giving rise to the issue commonly referred to as perceptual aliasing [2]. A robust VPR system should meet three essential criteria: condition invariance, viewpoint invariance, and generality, without the need for environment-specific VPR training [3]. Nevertheless, achieving both viewpoint and condition invariance simultaneously is a challenging endeavor.

The conventional handcrafted feature-based methods achieve good place matching performance in environments with slight viewpoint variations and illumination changes. Also, they are resource-efficient solutions in terms of computational cost. However, their performance degrades in extreme seasonal variations and highly dynamic environments.

In previous research [4], it has been demonstrated that holistic mid-layer features extracted from convolutional neural networks (CNNs) exhibit significant robustness against appearance variations (i.e., changes in conditions). However, they struggle to handle substantial changes in viewpoint. In contrast, some VPR approaches leverage techniques like bag-of-words [5,6] or VLAD [7,8,9] to aggregate local or regional features. These methods yield compact image representations and demonstrate considerable resistance to viewpoint variations. Yet, they overlook the spatial relationships between local features, making them prone to perceptual aliasing.

Conversely, the semantic information contained within images tends to remain relatively consistent. As a result, numerous studies have turned to leveraging semantic data to develop more resilient VPR methods. For instance, methods presented in [10,11] employ semantic segmentation boundaries for encoding images. However, it is important to note that the quality of segmentation can significantly affect the detection performance. On the other hand, a random walk approach is utilized in [12,13] to integrate semantic information into a three-dimensional graph. Although this approach captures comprehensive semantic information in images, it does come with the drawback of higher resource consumption. In practical applications, VPR methods must not only contend with a wide array of potential scene changes, including alterations in appearance and perspective, but also must meet the real-time demands of simultaneous localization and mapping (SLAM) systems. Therefore, it becomes crucial to design an efficient VPR method that can effectively handle the challenges.

**Figure 1:**
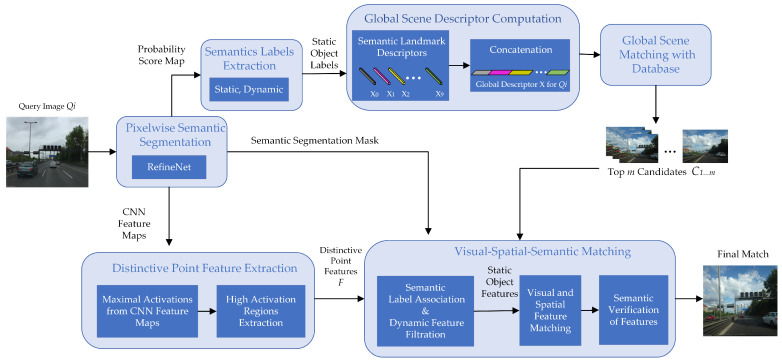
Working pipeline of the proposed semantic, visual, and spatial (SVS) information-aided hierarchical visual place recognition method.

In this research, we address the shortcomings of the existing literature and propose a place recognition method that leverages semantics-based global scene information along with the local visual information, shown in Figure 1, to handle the variations in both viewpoint and appearance and achieve robustness in such extremely dynamic environments. To achieve this, we harness semantics obtained from a state-of-the-art pixel-wise semantic segmentation network [14], alongside visual information extracted using convolution neural networks (CNNs). Our proposed method executes VPR through a stepwise, hierarchical matching approach. Initially, we conduct coarse matching that utilizes global scene semantics to compute the global semantic descriptors and perform matching, which condenses the search space and provides a list of potential match candidates. Then, in the fine matching phase, we identify the distinctive local features, extracted from CNN, and perform visual–semantic and spatial matching to pinpoint the true place match among the preselected candidates. Our approach demonstrates its effectiveness by achieving a meaningful recall at 100% precision. Moreover, our rationale for adopting this approach is rooted in the potential for a semantically aided system to enhance human communication and interaction with robots and vehicles.

The core contributions are as follows:

- A visual place recognition pipeline combining global scene semantics with appearance-based local correspondences.
- A robust feature extraction method is presented that extracts visually distinctive local features across all the feature maps obtained from the higher layer of CNNs. Such features possess scale- and viewpoint-invariant properties.
- A novel semantic visual and spatial information-based place matching method that utilizes distinctive local key correspondences between the image pairs for robust visual place recognition in extreme seasonal, light, and viewpoint variations.
- The SVS-VPR successfully attains a significant recall rate at 100% precision against state-of-the-art methods on challenging benchmark datasets.

The rest of the paper is structured as follows. In Section 2, we conduct a literature review to examine related work and establish the background for the problem. Section 3 outlines our proposed pipeline, while Section 4 details the experimental setup and results. Finally, in Section 5, we draw conclusions based on our findings and identify potential areas for future research.

## 2. Related Works

In the past decade, extensive research has been presented in the field of robot perception, primarily using camera sensors, in order to achieve autonomous navigation. This section presents a thorough taxonomy of the existing visual place recognition methods grouped into three major categories, i.e., appearance-based, deep learning-based, and semantics-based visual place recognition methods. Each of these categories is summarized along with its limitations in the subsequent sections.

### 2.1. Appearance-Based Methods

The conventional research on visual place matching predominantly relies on appearance-based handcrafted descriptors [15,16]. Notably, FAB-MAP [6] represents scenes using visual words and incorporates SURF [15] for detecting interest points. Nevertheless, a shared limitation of these handcrafted feature-based methods is the computational demands of the matching process. DBoW [17] addresses this issue by clustering feature descriptors using k-means, effectively reducing the complexity of matching and enhancing accuracy. However, it demands a considerable amount of time for dictionary training. Moreover, it suffers from performance degradation when the visual appearance of a place significantly differs in training and test images. To tackle these challenges, IBoW-LCD [18] aims to mitigate the problem by incrementally building the dictionary. Yet, in outdoor scenarios, substantial changes in appearance frequently result in the inadequacy of these methods.

Zaffar et al. [19] introduced an enhanced appearance-based technique, known as CoHOG, which utilizes a computationally efficient histogram of oriented gradients (HOG) image descriptor. CoHOG incorporates image entropy-based region of interest (ROI) extraction and region convolution matching methods, which help maintain performance in dynamic urban environments and conditional variations. However, along with the image representations, robust feature correspondence selection and matching play a significant role in correct place detection.

Recently, one such feature matching method has been presented in [20], where along with the appearance matching, spatial consistency is ensured for point features, ensuring the correct alignment of the match correspondences. Although the method successfully achieves improvement in place matching performance, it suffers from the non-detection of features in extreme seasonal variations and occlusion caused by the dynamic objects. Such limitations can be overcome by introducing semantics.

### 2.2. Deep Learning-Based Methods

With the advancement of deep learning networks across a wide spectrum of computer vision tasks, VPR has transitioned its emphasis towards the utilization of convolution neural networks to extract more comprehensive and versatile deep features.

Initially, Chen et al. [21] explored the use of convolutional neural networks in the context of VPR. Subsequently, CNNs have been applied in a range of studies, referenced as [22,23,24,25,26,27], all aiming to enhance the performance of VPR methods. For instance, Hou [24] applied the AlexNet [28] model to extract features and observed improved robustness in environments with notable illumination changes. In NetVLAD [7], a novel VLAD layer was integrated into the CNN architecture, leading to an end-to-end VPR approach that achieved impressive results. PatchNetVLAD [25,26] went a step further by optimizing NetVLAD and focusing on extracting local features. Meanwhile, RegionVLAD [9] extracted regional features from intermediate layers of pre-trained CNNs, offering computational efficiency as long as the environment remained unchanged. Nonetheless, it is important to note that CNNs necessitate extensive model training, and their accuracy is closely tied to factors like dataset size, variance within the dataset, and the nature of the training data.

Additionally, deep-learned representations of places often rely on global image descriptors for robust matching but tend to overlook the crucial spatial layout of images, which is vital for addressing perceptual aliasing. In order to handle this issue, distinctive landmarks have been extracted from images in [29], but encoding these landmark regions necessitates a visual vocabulary. Research presented in [30,31] trains a CNN to establish correspondences between images through geometric matching. Similarly, in [32], a Hypercolumn-based pixel-wise representation is employed for the precise localization of key points and object parts. Feng et al. [33] proposed a spatial and temporal information-based CNN feature-matching method that detects a place by first aligning them based on their spatio-temporal information. The STA-VPR performs well in environments with conditional variations. However, a common limitation in most of these methods is the lack of utilization of the pre-encoded semantic correspondence information embedded in convolutional neural networks, which leads to performance degradation in highly dynamic environments where most of the features are occluded due to dynamic objects and high viewpoint variations.

### 2.3. Semantics-Based Methods

The rapid evolution of dense pixel-wise semantic segmentation methods [14,34], driven by deep convolutional neural networks, has paved the way for the incorporation of semantic scene data into various facets of computer vision research. Likewise, semantic scene information holds significant importance in the domain of visual place recognition and localization, especially when it comes to matching images with extreme viewpoint variations. Recently, with the use of semantics, significant improvement in overall performance of place recognition has been reported. In [35], for instance, authors employ semantic masking to highlight categories with consistent appearances, like buildings, and generate an aggregated convolutional feature descriptor. However, this method requires environment-specific training. Similarly, in [36], semantic place categorization is used to enhance the performance of place recognition, particularly in diverse environments, but the focus is mainly on environment-related visual semantics. In [37], authors utilize geometric pairs of semantic landmarks designed for roadways to address environmental variations. While many of these methods primarily rely on semantic information to handle changes in appearance, in [12], a multi-view place localization system called X-view is introduced to deal with extreme viewpoint variations. Nevertheless, this approach relies solely on semantic labels and does not leverage appearance cues.

A different approach has been introduced in [38] that combines a visual feature graph extracted by a CNN with the semantic labels extracted from semantic segmentation, resulting in a powerful global descriptor that greatly improves VPR performance. This approach is particularly effective in matching locations under extreme variations in lighting and viewpoint, such as a 180-degree shift. In a similar vein, TNNVLAD uses two nearest neighbor local semantic tensors rather than relying solely on a single nearest neighbor match [39]. This algorithm enhances overall accuracy by comparing it with the descriptor of the second nearest neighbor.

Recently, several approaches have been developed that leverage semantic graph models to represent scenes using semantic segmentation outcomes, leading to more robust visual place recognition [40,41,42,43]. Authors of [40] use the Local Semantic Tensor in conjunction with semantic edge features extracted from semantic segmentation masks to detect correct place matches, while [41,42,43] generates topological connectivity graphs using pixel-wise semantic labels in the scene to match the spatial information. Though such techniques have shown improved performance compared to previous ones, it is important to note that the reported recall rate still falls short of achieving the desired performance, especially when dealing with extreme variations in environmental conditions.

Focusing on the limitations of the existing research and recognizing the role of deep features and semantics in handling challenges of visual place recognition, especially across varying environmental conditions and viewpoint changes, this research proposes the integration of pixel-wise semantic information with the deep feature-based visual information to improve the overall visual place matching performance, aiming for higher recall rate at 100% precision. We introduce a novel visual place matching method that can be generically applied to challenging environmental conditions, as presented in the following section.

## 3. Proposed Method

This section details the proposed semantics-aided hierarchical visual place recognition method, SVS-VPR. The overall pipeline is divided into two major steps, as depicted in Figure 1. Firstly, the global scene matching is performed using the scene semantics, which provides the match candidates while reducing the search space for the current image. Later, instead of using each CNN feature map as one feature, a robust feature extraction method is presented that extracts visually distinctive local point features across all the feature maps obtained from the higher layer of CNNs. Such features possess scale- and viewpoint-invariant properties. Further, a novel semantic–visual and spatial information-based place matching method is presented that utilizes distinctive local key correspondences between the image pairs for robust visual place recognition in extreme seasonal, light, and viewpoint variations.

### 3.1. Global Semantics-Based Scene Matching

#### 3.1.1. Semantics Extraction

For semantic information extraction, this research implements a Resnet-101-based RefineNet [14], a state-of-the-art multi-path refinement network for semantic segmentation of high-resolution images exploiting all the available information along the down-sampling process, which enables high-resolution prediction with long-range residual connections. It provides generic means to fuse coarse high-level semantic features with finer-grained low-level features to generate high-resolution semantic feature maps. The RefineNet is pre-trained on the Cityscapes dataset consisting of a sequence of images including indoor and outdoor environments. The dataset also exhibits seasonal, weather, light, and viewpoint variations, and dynamic objects. This research addresses the problem of visual place recognition in outdoor dynamic environments; thus, we extract only 20 semantic class labels belonging to the static and dynamic objects, as given in Table 1.

The semantic segmentation network outputs the semantic segmentation mask, including semantic class labels, at each pixel and probability score map containing probability scores for each class label within the map. The semantic segmentation mask has the same dimensions as the original image, while the probability score map is of size W×H×D. The width (W) and height (H) of each score map is ${\frac{1}{4}}^{th}$ of the original image I and depth (D) is equal to the number of semantic class labels.

In the semantic score map with *N* locations, each location *i* stores a D-dimensional descriptor $x_{i}$ containing probability scores corresponding to each semantic class, *s* where D=20. Thus, using maximum probability score *p* in $x_{i}$, the semantic class label can be assigned at each location *i*, as depicted in Equation (1)

(1) $$\begin{array}{cc} label_{i}=\underset{s}{argmax}\;p_{is} & \;s=1,...,20 \end{array}$$

#### 3.1.2. Global Scene Descriptor Computation and Matching

After the semantic label association with each pixel location, we compute the mean probability score μ for each class label using Equation (2).

(2) $$\mu_{s}=\frac{\sum_{i}^{N}\left\{x_{i}|label_{i}=s\right\}}{\sum_{i}^{N}\left\{i|label_{i}=s\right\}}$$

Here, *N* is the total pixel locations in a semantic score map. Aggregating the residual distribution from each semantic class *s* and the associated noise from the other class labels, the semantic descriptor $X_{s}$ is computed using Equation (3).

(3) $$X_{s}=\sum_{i}^{N}p_{is}\;\left|x_{i}-\mu_{s}\right|$$

Dynamic objects include the non-overlapping regions between the image pairs. Using those regions for place matching results in a high false detection rate. In order to avoid this, we generate the semantic aggregated semantic descriptors for semantic classes in the static objects category, as listed in Table 1, while excluding the dynamic objects. Finally, the semantically aggregated descriptors are concatenated to generate a global scene descriptor *X* for image I, as given in Equation (4).

(4) $$X=X_{0}+X_{1}+...+X_{9}$$

The global scene descriptor of each query image $Q_{i}$ is compared with *n* database images $D_{1,..,n}$ using the cosine function. The similarity between global semantic descriptor vectors $X_{Q_{i}}$ and $X_{D_{j}}$ from query $Q_{i}$ and database image $D_{j}$ is computed using Equation (5).

(5) $$S\left(X_{Q_{i}},X_{D_{j}}\right)=1-\frac{X_{Q_{i}}\cdot X_{D_{j}}}{∥X_{Q_{i}}∥\cdot ∥X_{D_{j}}∥}$$

The obtained global scene similarity scores *S* are further normalized to [0,1] using Equation (6).

(6) $$S_{Q_{i}}^{\prime }=\frac{S_{Q_{i}}-mean\left(S_{Q_{i}}\right)}{st.dev(S_{Q_{i}})}$$

For each query image $Q_{i}$, the database images $D_{1\ldots n}$ with the normalized global scene similarity score $S_{1...m}^{\prime }$ higher than the threshold α are given as global place match candidates $C_{1...m}$ to the local appearance-based place matching model, where m≤n.

### 3.2. Local Feature-Based Appearance Matching

Conventional image matching approaches that rely on handcrafted local features [44] initially identify distinctive keypoints within images and then apply robust descriptors for image representation [20]. Those descriptors are then matched through techniques such as RANSAC.

In contrast, image representations learned through deep neural networks for place recognition are typically global image descriptors. These descriptors inherently contain information about the entire visual content within the image, including distinctive local visual landmarks. Nevertheless, the specific local saliency information can be extracted from the neural network in various ways by utilizing the highly active regions within the convolutional feature maps. These active regions are commonly harnessed to create a more durable and dependable image representation [29,45].

Other than the existing methods, conventional methods extracting handcrafted point features, and CNN methods using feature maps or regions within feature maps as features, in this research, we present a unique method to extract the distinctive point features from the CNN feature maps and match those features in order to enhance the robustness of the place recognition against extreme environmental variations.

#### 3.2.1. Distinctive CNN Point Feature Extraction

**Figure 2:**
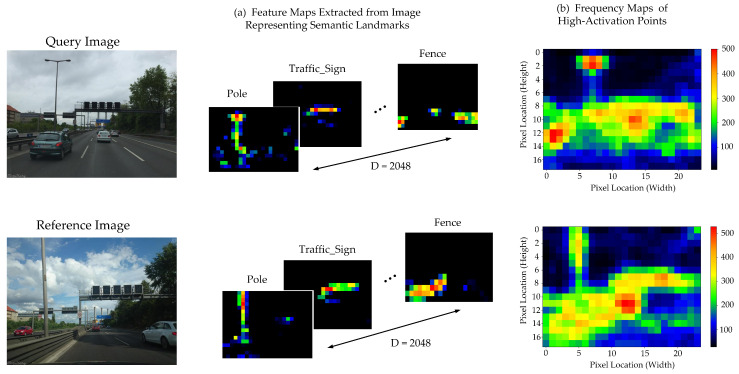
For an image pair, query image and reference image, (**a**) feature maps extracted from the neural network representing semantic landmarks and (**b**) high-activation regions computed across all the feature maps.

For local feature-based appearance matching, the CNN feature maps are extracted from the fifth convolutional layer of the RefineNet. The tensor width and height are 1/32th of the original image size, while the depth is 2048. Each feature map embeds activations representing the semantic landmarks in the image, as shown in Figure 2. Figure 2a illustrates feature maps extracted from the original query and reference images. The total 2048 feature maps extracted from an image contain the activations which correspond to any of the semantic landmarks among 20 classes.

These feature maps from perceptually similar images exhibit correspondence with each other. Thus, instead of matching the whole feature map of image pairs, we extract the maximal activations from each feature map as distinctive keypoints, which results in *N* number of keypoint features, where N=2048. It is important to notice that the number of keypoints *N* is considerably higher than the size (W×H) of the feature map, i.e., for Berlin A100, the feature maps extracted from the fifth layer of ResNet101-based RefineNet have size 24×18. Thus, we further reduce the number of extracted features by selecting only the highest activations at each pixel location, resulting in a maximum of features equal to the number of pixel locations in one feature map. Figure 2b illustrates a frequency map of maximally activated regions within an image *I* counted across all the 2048 feature maps. It is evident that only a few pixel locations have the tendency to stimulate activations across a wide array of feature maps.

Thus, we obtain the distinctive CNN point features *F* from each query image and database images for local appearance-based place matching, which is explained in the next subsection.

#### 3.2.2. Semantic–Visual–Spatial Matching

After the high-activation keypoint extraction, this module performs semantic, visual, and spatial information-based matching of each query image $Q_{i}$ with the list of candidate database images $C_{1...m}$ obtained as a result of global scene matching, explained in Section 3.1.2.

While matching the *i*th query image $Q_{i}$ and *j*th database image $C_{j}$, the semantic labels are first associated with the point features $F_{Q_{i}}$ and $F_{C_{j}}$ extracted from each of these images. This operation is performed to filter out the key activations from the dynamic objects while retaining only robust features from the static objects. It is essential to remove the dynamic features, as they include the non-overlapping regions from the image pairs, and matching scores computed based on such features result in high false detection rates. Moreover, filtering the dynamic features reduces the computation overhead that can be caused by directly matching all the features of image pairs.

For this purpose, we utilize the pixel-wise semantic segmentation mask obtained from the semantic segmentation network and resize them equal to the resolution of the feature map W×H, i.e., 24×18 in the case of Berlin A100. Using the (x,y) coordinate location of each keypoint in the feature map, its semantic label can be extracted from the semantic segmentation mask. Let *a* be the point feature in $F_{Q_{i}}$ with (x,y) coordinates in feature map *M*. Its semantic label $l_{a}$ from the semantic segmentation mask SemMask of $Q_{i}$ can be extracted using the (x,y) coordinates, as demonstrated in Equation (7)

(7) $$l_{a}=Q_{i}\left(SemMask\left[x\right]\left[y\right]\right)$$

Using the above equation, the semantic labels for all the keypoints in $F_{Q_{i}}$ and $F_{C_{j}}$ are extracted, represented as $L_{Q_{i}}=l_{1...a...n}$ and $L_{C_{j}}=l_{1...b...n}$, where $L_{Q_{i}}$ and $L_{C_{j}}$ are the set of semantic labels for the keypoint correspondences in $Q_{i}$ and $C_{j}$. The semantic labels are the integer values, listed in Table 1. Here, we keep the features only from the static object category, while discarding those from the dynamic objects. This step effectively eliminates a large number of activations, particularly those linked with random activations triggered by the convolutional filters detecting features that are not present in the specific regions, and provides a set of robust features to be matched between image pairs.

In the next step, we perform the visual and spatial matching of these point features. As explained in Section 3.2.1, each point feature is the maximal activation, at a specific pixel location, across all the feature maps extracted from an image. Thus, each feature is a D-dimensional feature vector, where D is the depth of the tensor, i.e., 2048. The feature vectors of the query image are matched with those of the database image. Let *a* be the D-dimensional feature vector from the query image $Q_{i}$ and *b* be the D-dimensional feature vector from the candidate database image $C_{j}$. The visual matching between both of these vectors is performed by using the cosine distance function [46], as depicted in Equation (8).

(8) $$V\left(a,b\right)=1-\frac{\sum_{k=1}^{D}\left(a_{k}\cdot b_{k}\right)}{∥a∥\cdot ∥b∥}$$

The match correspondences between the image pair are then spatially verified. For this purpose, a neighborhood window *N* of size w×h is used to limit the spatial displacement of the features. Thus, for a feature vector *a* with x,y location in the feature map of $Q_{i}$, the feature vector *b* with $x^{\prime },y^{\prime }$ location in the feature map of $C_{j}$ is considered as a true spatial correspondence if $b(x^{\prime },y^{\prime })$ lies within the neighborhood window where a(x,y) is the center of the window. In this paper, we have used the window size of 3×3. This spatial matching facilitates the avoidance of false positives caused by perceptual aliasing while facilitating the match correspondences in large viewpoint changes.

After filtering the spatially consistent match pairs, semantic label verification is performed to further refine the matching performance. The semantic labels, computed using Equation (7), of each feature match pair are compared. The visually and spatially consistent feature match correspondences with the same semantic label are retained, while feature pairs that are semantically inconsistent are discarded. Equation (9) illustrates the semantic label matching for the visually and spatially consistent match pair (a,b) among a list of all feature match pairs *P*, where *a* and *b* are the key features from $Q_{i}$ and $C_{j}$.

(9) $$P\left(a,b\right)=\left\{\begin{array}{cc} 1, & if\;l_{a}=l_{b}\\ 0, & otherwise \end{array}\right.$$

We normalize the cosine distance, obtained using Equation (8), for the remaining feature match pairs using L1-normalization and subtract the maximum distance value from each of them for normalization across all the match pairs, as shown in Equation (10).

(10) Distance[k]=Max(normDistance)−normDistance[k]

Here, normDistance[k] is the L1-normalized distance between $k^{th}$ match pair, while Distance is a vector storing the distance values between feature pairs of $Q_{i}$ and $C_{j}$ across the visually, spatially, and semantically consistent feature pairs.

Finally, a weighted distance value is computed between the feature pairs in *P* of query $Q_{i}$ and candidate image $C_{j}$ using Equation (11).

(11) $$Score_{C_{j}}=\frac{cosine\_distance(P_{Q_{i}}*Distance,P_{C_{j}}*Distance)}{∥Distance∥}$$

$P_{Q_{i}}*Distance$ and $P_{C_{j}}*Distance$ represent element-wise multiplication of the vectors $P_{Q_{i}}$ and $P_{C_{j}}$ with the Distance. The cosine distance is computed between the two resulting vectors and is divided by the Euclidean norm of the Distance vector. The $Score_{C_{j}}$ is the place matching score obtained for the *j*th candidate image. The candidate image with the lowest score value is considered the final place match.

## 4. Experimental Results

### 4.1. Implementation Setup

The proposed method is implemented and evaluated on an Intel(R) Core (TM) i-10900X CPU running at 3.70 GHz. The semantic segmentation is performed on an RTX 3090 GPU. The feature extraction and matching are performed using Python 3.

### 4.2. Datasets

This research aims to develop a visual place recognition method while handling the challenging environmental variations. For this purpose, the proposed algorithm is evaluated on three publicly available benchmark datasets: Oxford Robotcar [47], Mapillary [29], and Synthia [48]. Collectively, these datasets exhibit strong seasonal, weather, illumination, and viewpoint changes in dynamic environments. Moreover, the data acquisition method also varies significantly, thus making these datasets even more challenging for achieving place recognition. The key information of these datasets is mentioned in Table 2. Further details are as follows:

#### 4.2.1. Mapillary

It is a crowd-sourcing platform providing several datasets. From Mapillary, we have used the Berlin A100 and Berli Kudamm datasets. Both of these datasets were acquired in an urban environment. Each comprises two image sequences captured along the same route by different users exhibiting very large viewpoint changes and highly dynamic environments. A large number of dynamic objects causing occlusion to the static features results in a lack of essential feature detection and matching. To establish ground truth, we utilized the geotagged data from the dataset, with a frame tolerance in the range of ±3 frames.

#### 4.2.2. Oxford Robotcar

It is a large dataset recorded in Oxford City and consists of a number of traverses across the year exhibiting seasonal, illumination, and viewpoint variations. For evaluation of the proposed method in terms of seasonal variations, Summer (2015-05-19-14-06-38), Winter (2015-02-03-08-45-10), and Autumn (2014-12-09-13-21-02) traverses have been used, while Day (2014-12-16-09-14-09) and Night (2014-12-10-18-10-50) traverses are used for performance analysis during light changes. Along with conditional and viewpoint variations, this dataset also has the characteristics of motion blur and overexposure, making the pace recognition an even more challenging problem. Each traverse is sampled selecting one frame every 2 m using GPS data. Ground truth information is established based on GPS data, with a match considered a true positive if it falls within a 10 m range, following a thresholding approach similar to [49,50].

#### 4.2.3. Synthia

SYNTHIA provides a synthetic dataset of urban scenes. In this research, we have used the image traverses from Sequence 2 where Spring, Summer, and Winter image sequences are used to evaluate the robustness of the proposed method against seasonal variations, while Dawn and Night traverses are used for evaluation in extreme light changes. All of these traverses are approximately 1.5 km in length. Ground truth is computed using GPS data, where a match is considered a true positive if it falls within a 10 m range.

### 4.3. Ablation Study

#### Global Scene Similarity-Based Candidate Selection

**Figure 3:**
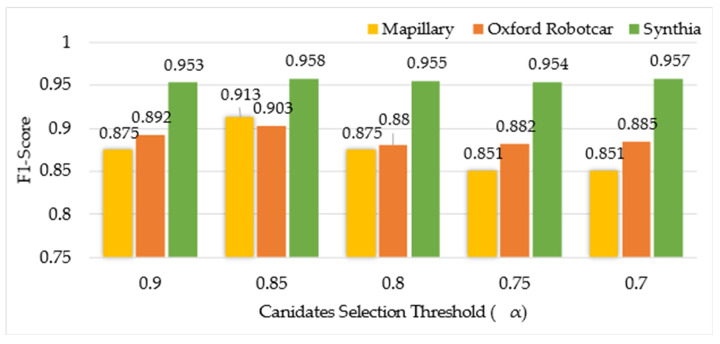
F1-score obtained using global semantics-based scene matching when different values of candidate selection threshold (α) are used.

**Figure 4:**
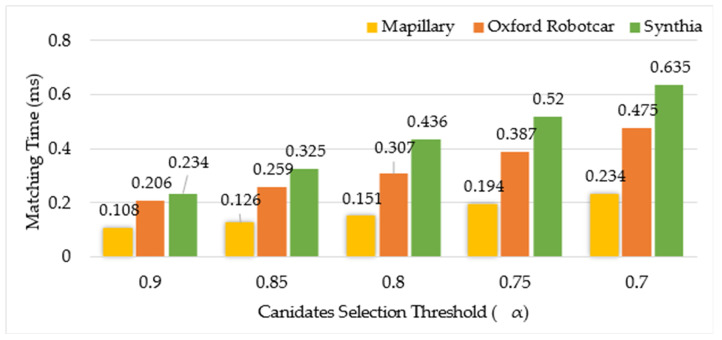
Matching time comparison for different values of threshold α.

To analyze the influence of the candidates selected based on the scene similarity score on the whole method, F1-score and matching time have been adopted as the performance indicators. Note that matching time here refers to the time of local CNN feature-based place matching performed after the selection of candidates based on different values of α. We select the number of candidates based on the global similarity score threshold set to 0.9, 0.85, 0.8, 0.75, and 0.7, respectively. The results of matching time and F1-Score are shown in Figure 3 and Figure 4.

As can be seen from Figure 3, for each dataset, there is little difference in the F1-Score on each dataset for different threshold values.

The results in Figure 4 show that matching time of the Mapillary dataset is lower than that of the Oxford robotcar and synthia datasets on the whole. This is because the size of the two datasets is significantly different.

Moreover, the matching time increases greatly when the number of candidates is increased due top low threshold values.

Taking account of matching time and F1-score, we find that the effectiveness is better when the similarity threshold α is set to 0.85. Finally, in the comparison experiment, we used α=0.85.

### 4.4. VPR Performance Analysis

This section presents the experiments conducted to evaluate the performance of the proposed method in comparison to the state-of-the-art CNN feature-based visual place recognition methods, namely NetVLAD [7] (VGG-16 + NetVLAD + whitening, Pittsburgh), LoSTX [38] and STA-VPR [33]. NetVLAD is a viewpoint-robust CNN model providing end-to-end visual place recognition and is able to achieve great performance on most datasets. STA-VPR is also a CNN feature-based VPR method using spatial information for feature alignment and matching. LoSTX is the semantics-enabled place recognition method providing high robustness with the use of semantics. All of these methods are implemented by running their open-source code in the default configuration provided by the authors.

**Figure 5:**
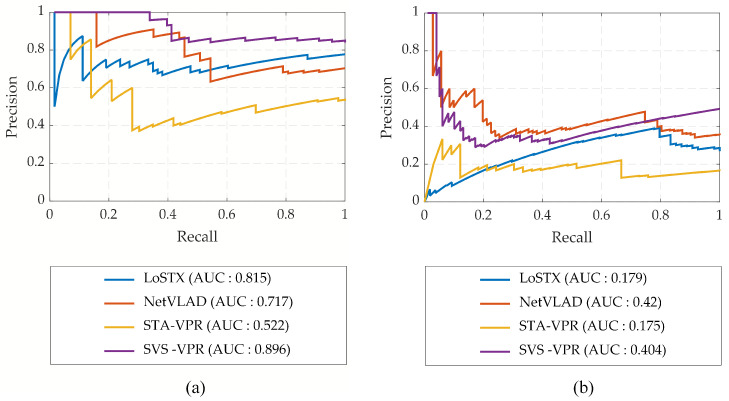
Precision–recall curves obtained for visual place recognition on Mapillary dataset: (**a**) Berlin A100; (**b**) Berlin Kudamm.

Figure 5a,b illustrate the precision–recall curves computed for Berlin A100 and Berlin Kudamm datasets acquired from Mapillary. These datasets pose significant challenges, including drastic changes in viewpoint, moderate variations in illumination, and dynamic objects causing feature occlusion. In the Berlin A100 dataset, both the reference and query traverses were recorded from a car, leading to substantial lateral and slight angular viewpoint changes. In the Berlin Kudamm dataset, the reference data images were recorded from the bus deck, and the query traverse was captured from the car dashboard. The Mapillary dataset is particularly demanding due to the presence of dynamic objects and non-overlapping regions, making it difficult to identify robust features for effective place matching. It is worth noting that most other methods struggle to provide practically useful results in these demanding datasets. In contrast, the proposed method demonstrates a remarkable performance, achieving the highest recall rate at 100% precision in Figure 5a,b. Furthermore, the area under the precision–recall curve is also mentioned. SVS-VPR outperforms the other methods on the Berlin A100 dataset, as shown in Figure 5a, while on the Berlin Kudamm dataset, NetVLAD has shown higher AUC than SVS-VPR.

**Figure 6:**
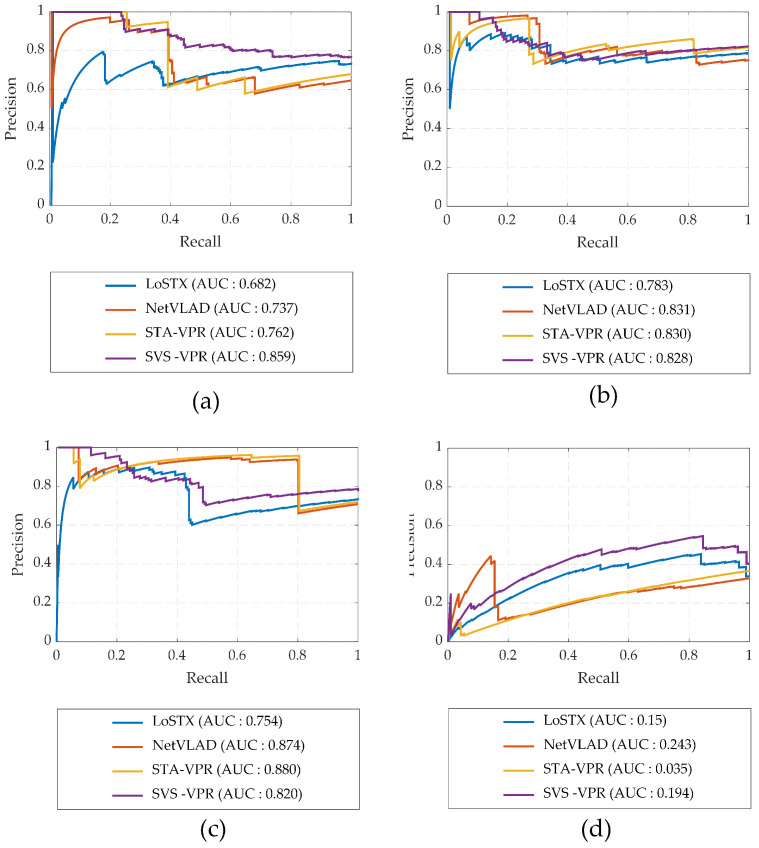
Precision–recall curves for visual place recognition using different traverses of Oxford RobotCar dataset: (**a**) Summer–Autumn; (**b**) Summer–Winter; (**c**) Autumn–Winter; and (**d**) Day–Night.

Figure 6 demonstrates the comparative performance of the proposed method relative to the state-of-the-art visual place recognition methods on the Oxford RobotCar dataset. This dataset presents notable challenges due to substantial seasonal variations, weather fluctuations, and shifts in illumination. In Figure 6a, it is observed that using the Summer traverse as a reference dataset results in a higher recall rate and AUC when compared to the Autumn traverse. This is attributed to the relatively minor environmental changes during the summer season. However, in Figure 6b, the recall rate decreases when the Summer traverse is compared with the Winter traverse, mainly due to the extreme seasonal variations causing significant alterations in the environmental appearance. Additionally, variations in weather conditions, such as sunny conditions in the Summer traverse versus overcast weather in the Winter and Autumn traverses, contribute to illumination differences alongside seasonal changes. Figure 6c highlights that employing the Autumn traverse as a reference dataset when matched with the Winter traverse leads to improved place matching results. Despite substantial seasonal variations between the two traverses, the similarity in weather conditions (e.g., overcast skies) and consistent environmental factors (e.g., few or no leaves on trees and absence of shadows) enhances matching performance. In Figure 6d, the VPR performance with the Day traverse as a reference dataset and its comparison with the Night traverse is showcased. This dataset is particularly demanding due to extreme illumination variations. The proposed method surpasses other state-of-the-art techniques, achieving a remarkable recall rate at 100% precision in this challenging scenario. In spite of the higher recall rate, the extreme seasonal and illumination variations affect the performance across the entire dataset, resulting in comparatively lower AUC obtained by SVS-VPR than other methods.

**Figure 7:**
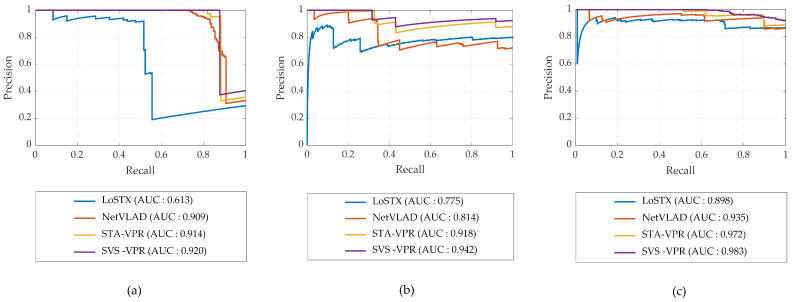
Precision–recall curves obtained for visual place recognition on Synthia dataset: (**a**) Spring–Summer; (**b**) Spring–Winter; and (**c**) Dawn–Night.

Similarly, when applied to the Synthia dataset, the proposed method consistently outperforms other approaches, demonstrating a remarkable recall rate, as depicted in Figure 7. Figure 7a,b present the precision–recall results obtained by using image sequences from the Spring traverse as the reference database and those from Summer and Winter for matching. This dataset presents challenges such as frequent turns and the presence of highly dynamic objects like pedestrians and vehicles, along with a homogenous scene that leads to perceptual aliasing, making it a demanding dataset. Nevertheless, the proposed method excels in achieving superior results. Figure 7c showcases the place matching outcomes from Dawn and Night traverses. Notably, in Figure 7c, the performance of all place recognition methods is significantly improved. This improvement is attributed to the fact that both the Dawn and Night traverses exhibit nearly identical illumination conditions, facilitating more effective matching.

Table 2 demonstrates the quantitative comparison of Sem-VPR and the SOTA methods in terms of true place detection rate among the total detections (%). Sem-VPR outperforms the best-performing CNN feature-based methods, NetVLAD and STA-VPR, and semantics-aided CNN-based VPR method, LoSTX, respectively. The differences are particularly pronounced in datasets with large appearance variations, i.e., Oxford Robotcar and Synthia (both seasonal changes and including images captured at night time) and Mapillary (very large viewpoint variations).

### 4.5. Computational Cost Analysis

Computation time for visual place recognition is of vital importance in real-time robot applications. Considering the real-time application of the proposed algorithm, the processing pipeline can be divided into the offline and online mode, where semantics and CNN feature extraction from the database images is performed in the offline mode, while query image matching is performed in the online step. Table 3 shows the mean processing time (s) per query for online steps involved in the proposed visual place recognition algorithm pipeline. The semantic labels extraction, global scene descriptor computation, and matching are included in the global semantics-based scene matching. The local feature-based appearance matching involves the computation time for distinctive local feature extraction and robust feature matching using visual, semantic, and spatial information. It can be observed that the semantic segmentation using RefineNet is computationally expensive. However, using the high computational power, the computation time can be significantly reduced. Also, it is not an essential requirement and based on the application, other dense semantic segmentation networks can also be adopted, giving real-time performance [51]. The proposed SVS-VPR algorithm takes the semantic segmentation output from the semantic visual SLAM system, which handles the pixel-wise semantic segmentation in a parallel thread, as input and performs the feature extraction and visual place recognition for the query image. Collectively, the computation time for global and local information-based visual place recognition is less than 0.3 milliseconds, which meets the real-time requirements.

Table 4 presents the comparison of computation time for SVS-VPR with the state-of-the-art algorithms. The execution time includes the time required for feature extraction from the query image and time required for query image matching with the database. The proposed method succeeds in achieving real-time computational performance for feature encoding and visual loop closure detection in comparison with the SOTA methods.

## 5. Conclusions

This study introduces a novel method called SVS-VPR for visual place recognition for visual Simultaneous Localization and Mapping (SLAM) systems. The core concept is to combine global scene semantics-based place matching with local information based on visual, spatial, and semantic cues to achieve comprehensive place identification that incorporates both local and global context. SVS-VPR combines appearance-based matching with semantics matching to enhance the overall performance of place detection. The method is evaluated using benchmark datasets that encompass a range of environmental conditions, including seasonal changes, variations in viewpoint, and shifts in illumination in dynamic urban settings. The experimental results clearly indicate that SVS-VPR offers higher detection accuracy and improved robustness when compared to existing approaches. This research excels in scenarios with extreme seasonal changes and minor viewpoint variations within highly dynamic urban environments. However, recognizing places in situations with extreme illumination variations from day to night remains a challenge. Furthermore, there is limited attention given to place recognition under adverse weather conditions, such as rain and fog, both during the day and night. As part of our future work, we intend to expand our research to address the challenges posed by extreme illumination variations and adverse weather conditions, aiming to further enhance the capabilities of our approach. 

## Figures and Tables

**Table 1:** Semantic class labels extracted from semantic segmentation network.

Category	Semantic Classes	Labels
Static Objects	Road, Building, Vegetation, Pole, Traffic_Light, Traffic_Sign, Terrain, Wall, Fence, and Sidewalk	0, 1, ..., 9
Dynamic Objects	Sky, Person, Rider, Car, Truck, Bus, Train, Motorcycle, Bicycle, and Void	10, 11, ..., 18, 255

**Table 2:** True place detection rate (%) obtained for Sem-VPR in comparison to other SOTA methods on benchmark datasets.

Methods	Mapillary		Oxford RobotCar				Synthia		
	**Berlin** **A100**	**Berlin** **Kudamm**	**Summer-** **Autumn**	**Summer-** **Winter**	**Autumn-** **Winter**	**Day-** **Night**	**Spring-** **Summer**	**Spring-** **Winter**	**Dawn-** **Night**
NetVLAD	0.74	0.35	0.65	0.75	0.71	0.33	0.31	0.72	0.86
LoSTX	0.78	0.27	0.73	0.78	0.73	0.34	0.29	0.79	0.87
STA-VPR	0.53	0.16	0.69	0.81	0.72	0.37	0.36	0.88	0.89
Sem-VPR (ours)	**0.84**	**0.49**	**0.76**	**0.82**	**0.77**	**0.41**	**0.40**	**0.92**	**0.91**

**Table 3:** Mean execution time (ms) per query image for each module of the proposed Sem-VPR on benchmark datasets.

Components of Proposed Method	Mapillary	Oxford Robotcar	Synthia
Pixel-wise Semantic Segmentation	468.9	464.9	620.2
Global Semantics-based Scene Matching	0.027	0.029	0.041
Local Feature-based Appearance Matching	0.107	0.258	0.242

**Table 4:** Mean execution time (s) per query image for benchmark dataset.

Methods	Mapillary	Oxford Robotcar	Synthia
NetVLAD	0.910	1.953	1.42
LoSTX	0.685	1.052	1.491
STA-VPR	0.557	0.702	0.888
SVS-VPR	0.469	0.465	0.620

## Data Availability

Data are contained within the article.
